# Bioecological Drivers of Rabies Virus Circulation in a Neotropical Bat Community

**DOI:** 10.1371/journal.pntd.0004378

**Published:** 2016-01-25

**Authors:** Benoit de Thoisy, Hervé Bourhy, Marguerite Delaval, Dominique Pontier, Laurent Dacheux, Edith Darcissac, Damien Donato, Amandine Guidez, Florence Larrous, Rachel Lavenir, Arielle Salmier, Vincent Lacoste, Anne Lavergne

**Affiliations:** 1 Laboratoire des Interactions Virus-Hôtes, Institut Pasteur de la Guyane, Cayenne, French Guiana; 2 Lyssavirus Dynamics and Host adaptation Unit, National Reference Centre for Rabies, Institut Pasteur, Paris, France; 3 Sylvétude, Office National des Forêts, Cayenne, French Guiana; 4 Laboratoire de Biométrie et Biologie évolutive, UMR CNRS 5558, Université Lyon 1 / CNRS, Villeurbanne, France; Armed Forces Health Surveillance Center, UNITED STATES

## Abstract

**Introduction:**

In addition to the commonly accepted importance of the vampire bat in the maintenance and transmission of the rabies virus (RABV) in South America, RABV infection of other species is widely evidenced, challenging their role in the viral cycle.

**Methodology / Principles findings:**

To identify the bioecological drivers of RABV circulation in neotropical bat communities, we conducted a molecular and serological survey on almost 1,000 bats from 30 species, and a 4-year longitudinal survey in two colonies of vampire bats in French Guiana. RABV was molecularly detected in a common vampire and in a frugivorous bat. The sequences corresponded to haematophagous bat-related strains and were close to viruses circulating in the Brazilian Amazon region. Species’ seroprevalence ranged from 0 to 20%, and the risk of seropositivity was higher in bats with a haematophagous diet, living in monospecific colonies and in dense forests. The longitudinal survey showed substantial temporal fluctuations, with individual waves of seroconversions and waning immunity. The high prevalences observed in bat communities, in most habitats and in species that do not share the same microhabitats and bioecological patterns, the temporal variations, and a rather short period of detectable antibodies as observed in recaptured vampires suggest (i) frequent exposure of animals, (ii) an ability of the infected host to control and eliminate the virus, (iii) more relaxed modes of exposure between bats than the commonly assumed infection *via* direct contact with saliva of infected animals, all of which should be further investigated.

**Conclusions / significance:**

We hypothesize that RABV circulation in French Guiana is mainly maintained in the pristine forest habitats that may provide sufficient food resources to allow vampire bats, the main prevalent species, to survive and RABV to be propagated. However, on the forest edge and in disturbed areas, human activities may induce more insidious effects such as defaunation. One of the ecological consequences is the disappearance of resources for tertiary or secondary consumers. Populations of vampires may then shift to alternative resources such as cattle, domestic animals and humans. Therefore, a good forest status, allowing both a dilution effect in highly rich bat communities and the maintenance of large populations of medium-sized and large mammals used as prey by vampires, should prevent their migration to anthropized areas.

## Author Summary

The vampire bat is known to be the main reservoir of the rabies virus (RABV) in South America. Nevertheless, other bat species are implicated in the cycle of the virus. Indeed, seven genus-specific rabies lineages have been described in insectivorous bats in Brazil. In French Guiana, we looked for the presence of the virus in a large number of bats, belonging to 30 different species. We found a high rate of seropositive animals, mainly haematophagous bats, and in those living in monospecific colonies and in forest habitats. We also monitored two colonies of vampire bats over a 4-year period and found that some animals became seropositive for the RABV, while others, after being seropositive, were able to become seronegative. These data first of all demonstrate that the virus widely circulates in bat communities with transmission occurring *via* direct saliva contact with broken skin and mucosa at the intra-specific level in vampire bat species. Exposure of other bat species, including those that do not share the same microhabitat, occurs in all forest strata through modes of transmission that have yet to be determined. Secondly, these animals seem to be exposed regularly, and most of them have a great ability to control and eliminate the virus. Third, these results suggest that pristine forest habitats provide sufficient food resources for the survival of vampire bats and propagation of RABV. In contrast, in disturbed habitats, where resources are decreasing, the vampires might have to shift to alternative resources such as cattle, domestic animals or even human beings. Altogether, the risk of rabies virus transmission may increase on the edge between forest and anthropized areas.

## Introduction

Rabies is recorded in many Latin American and Caribbean countries where it was responsible for 111 lethal human cases between 2010 and 2012 [1]. Two epidemiologic cycles are recognized. The urban cycle involves carnivores, most particularly dogs, as the main reservoir and considerable effort to vaccinate and control stray dogs has made it possible to move from endemicity to episodic events in circumscribed areas [2]. The sylvatic cycle, involving the common vampire bat *Desmodus rotundus* as the major reservoir among wild species [3,4], shows an increasing number of cases over time [5]. Contrasting with the urban cycle, the sylvatic cycle is considered as endemic in South America, with emergence of local foci restricted in space and time correlated with surveillance investigations after outbreaks in humans and/or domestic animals [2]. In the Americas, bats are now considered as the principal rabies reservoirs [6]. Despite extensive monitoring and case studies [7], the ecological factors that could influence the frequency and outcome of cross-species transmission have not been sufficiently investigated. A global analysis performed on bat communities in North America highlighted that cross-species transmission is mainly associated with phylogenetic relatedness and geographic overlap between species and to a lesser extent to ecological factors [8]. Nevertheless, the role of ecological factors in cross-species transmission was not yet investigated in tropical environment.

Recent extensive serological surveys have shown a high diversity of positive bat species, suggesting a wide distribution and circulation of the virus in Amazonia [2,9–12]. However, contrasting with serological surveys, necessary to qualify the emergence risk, molecular surveys have been hitherto scarce. Most of them focused on previously recognized reservoirs such as the *Desmodus rotundus* [4]. Nevertheless, in Peru, rabies virus (RABV) was also found in *Histiotus montanus* [13,14] and *Artibeus lituratus* [15]. In the Southern cone of South America, several species have been shown to be reservoirs of distinct lineages of rabies, including *Tadarida* sp., *Artibeus lituratus*, *Eptesicus furinalis*, *Lasiurus ega* and *L*. *blossevellii*, *Myotis nigricans* and *Molossus molossus*, for example [16–18]. These studies suggest that non-haematophagous bats could also act as primary hosts of the virus [2]. Nevertheless, their ability to spread the virus among populations requires further attention. Rather than focusing on some species, bat communities must therefore be considered as a whole in order to understand the diversity of hosts, dispersal and dynamics of viral strains, and the likelihood of their emergence.

The ability of bats to control rabies virus (RABV) infection and to disperse the virus, inter-individual transmissions as well as viral persistence, are other points that remain to be examined. Epidemics of rabies are often reported with wave-like patterns, both geographically [19] and temporally [20]. Several key factors have been suggested for viral maintenance and cross-species transmission in bat colonies: (1) a spatial process involving rabies infection in a “naive population” by individuals that migrate from neighbouring colonies and frequent immunization of bats [21]; (2) regional enzootic maintenance correlated with frequent independent transmission events and the major role played by naive juvenile and sub-adult populations in viral maintenance [7,22]; (3) long incubation period associated with a low mortality of bats during hibernation in subtropical areas and temperate areas, which allow enough infected individuals to survive until the following year and to infect the new generation of susceptible young [23]; (4) the effects of climate on the viral evolution of bat rabies across temperate and tropical regions with a major impact on cross-species transmission [24]. For the common vampire bats, experimental infections suggest that they are quite resistant to infection and that only a high viral dose induces symptoms [25] with alteration of behaviour and neurological signs [26]. Furthermore, fields and statistical studies indicate that the probability of a vampire bat developing a lethal infection with rabies virus is low (around 10%) [21]. Transmission between individuals is classically believed to be based on close interactions, both intra- and interspecific, facilitated, for instance, by shared roosts, regurgitation of blood meals, and social licking, with saliva in contact with broken skin as the main transmission route of the virus [27,28]. However, growing evidence of a wide distribution of seropositivity in species with distinct bioecological patterns [2,11,12,29] raises the question of which mechanisms are implicated in cross-species transmission.

Lastly, emergence foci are challenging to predict, and thereafter to control, in time and space. Biodiversity plays a key role in the transmission of zoonotic diseases. To date, no correlation has been observed between bat species richness in Latin America and the number of rabies-positive species. Nevertheless, this observation was limited by the programmes implemented and the ability of the different countries to investigate rabies virus circulation [2]. The debate remains active on the relative and contradictory importance of the dilution effect, claiming that loss of diversity would favour the risk of emergence as well as the amplification effect, which assumes a positive correlation between biodiversity and disease risk [30]. Rabies occurrence has been reported in the Brazilian state of Pará, in Ecuadorian and Peruvian Amazonia, as well as in Colombia and Trinidad. These areas share a similar environment pattern: they have experienced substantial forest loss during the last few decades [31], likely associated with significant modifications and disturbances of their wild animal communities, including modification of bat communities as classically observed in disturbed habitats [32]. Extensive cattle ranching also provide stable resources and can results in population expansion for vampire possibly associated with rabies incidence [21,22].

In the Amazonian region, the number of human rabies cases remains high in spite of efforts undertaken in education, prevention, vaccination and in bat population control. Numerous epidemics in Amerindian populations have been reported in Peru [5,33]. Between 1996 and 2010, 113 human rabies cases associated with vampire bats were registered in Peru and in 2011, eight children died from rabies in an Amerindian community [33,34]. In Brazil, numerous cases have also been reported in the Amazonian region with more than 62 persons infected between 2004 and 2005 [35,36]. In French Guiana, a French overseas department located on the northern part of the Amazonian region, 13 rabies cases were recorded in cattle and domestic carnivores between 1984 and 2011 [37]. They were all related to vampire bat-associated rabies virus. In 2008, a human rabies case was recorded. The origin of the contamination was not formally established but transmission by a vampire bat bite was favoured [38], molecular typing of this virus (polymerase (L) gene, Genbank accession number GU816007) showed a close relationship with the viruses circulating in haematophagous bats in neighbouring countries.

The aim of this study was to better understand bioecological factors associated with virus circulation within different bat communities and environments. More specifically, we aimed to (i) investigate how factors related to the environment (season, vegetation type, biogeographic region, disturbance level) and to hosts (diet, social structure, type of roosts), could explain the variations in rabies prevalence; (ii) explore the maintenance of the virus in colonies and (iii) examine how the observed patterns inform on the circulation, transmission modes and emergence risks. To achieve these aims, we implemented serological and molecular surveys on wild bats from French Guiana, on a wide diversity of sites and bat communities, and through a 4-year serological temporal monitoring of two free-ranging vampire bat colonies.

## Materials and Methods

### Ethical and legal statements

Animals were captured, handled, sampled and, whenever necessary, euthanized in accordance with ASM guidelines [39], under the supervision of researchers in possession of the national "expérimentation animale level 1" degree. Bats are not protected by law in French Guiana, but the project was presented to the Conseil Scientifique Régional pour le Patrimoine Naturel de la Guyane, and approved by this council. Captures that occurred within protected areas (nature reserves) received approval of the Conseil Scientifique Régional du Patrimoine Naturel the 26^th^ January 2010 and ad-hoc authorizations (no. 2011–35 from the 05/30/2011, no. 35 and 59 obtained 03/21/2013 and 04/17/2013, respectively, and delivered by the Préfecture de la Guyane). Sera were exported from French Guiana to the National Reference Center (NRC) for Rabies, Institut Pasteur, Paris, France, for serology, under authorization no. 141/DEAL/2013 delivered by the Préfecture de la Guyane.

### Study areas

**Fig 1:**
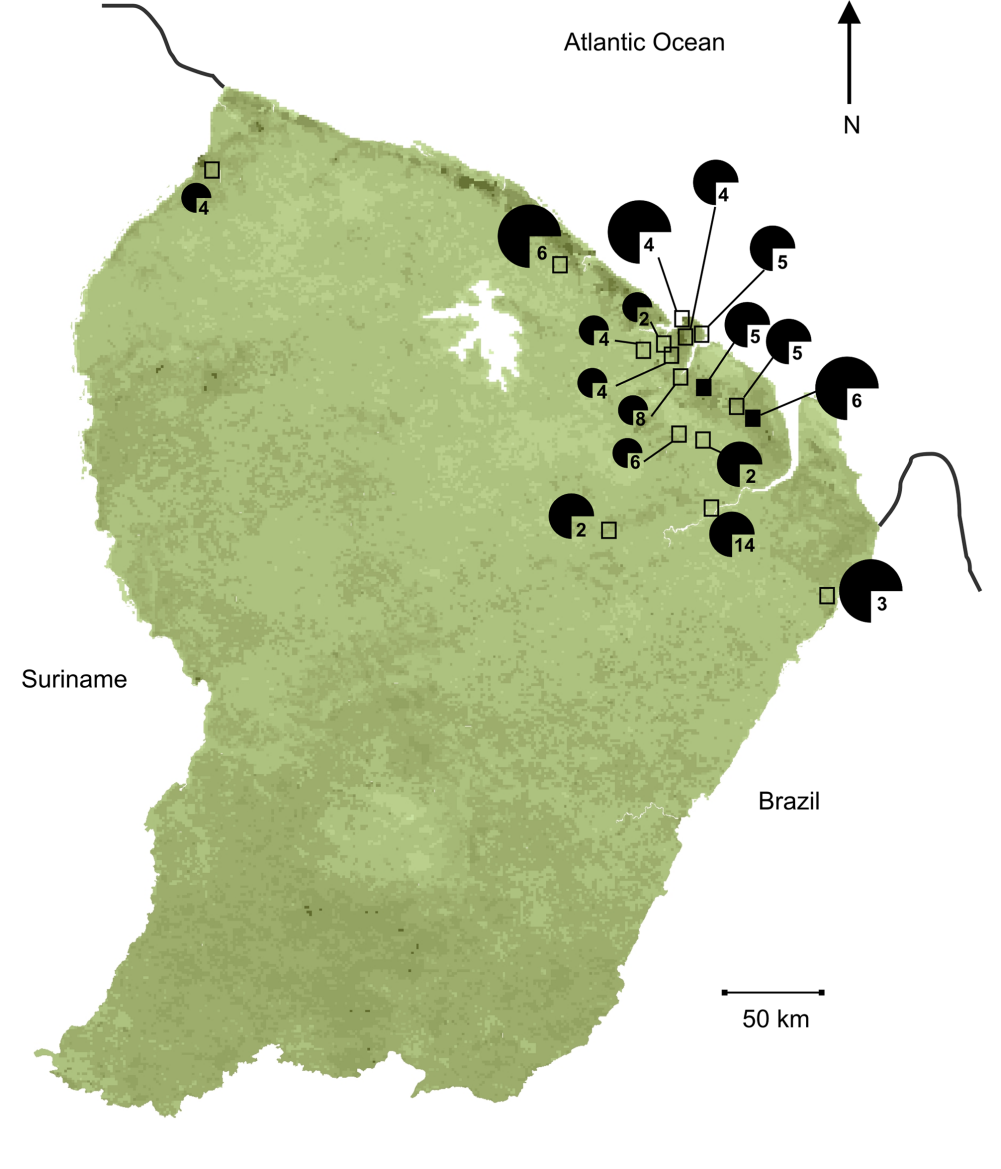
French Guiana map with the location of the trapping sites in empty squares and the caves (cave 1 and cave 2) in black squares investigated in the study. For clarity, close sites (<10 km) are presented together. Pie chart indicates rabies seroprevalence, with small charts indicating prevalence rates < 5%, medium-sized, charts prevalence rates from 5 to 15%, and large charts, prevalence rates > 15%. The number associated to the chart indicates the number of species sampled at the trapping site. Background of the map shows the vegetation type (Gond et al. 2011), the main environmental variable associated to the variation of the seroprevalence.

Bats were trapped in 24 sites from 2005 to 2013 (Fig 1, Table 1, S1 Table). These sites were located in pristine primary lowland forests with low disturbance pressures (six sites), edge habitats (five sites), anthropized areas (eight sites) and urban and periurban areas (five sites). The sites’ disturbance level was determined with the human footprint method [40], based on both evidenced pressures on natural habitats (density of roads and tracks, logging, mining) and signs of human presence (settlements). Forest types follow the classification based on remote-sensing analysis of reflectance of the vegetation cover [41], biogeographic regions were identified from geomorphology and large vegetal communities [42]. Longitudinal surveys of vampire bats were conducted in two breeding caves with the Marais de Kaw-Roura Nature Reserve.

**Table 1:** Bat species tested for rabies serology, prevalence, and specific bioecological factors.

Family and species of bats	N tested	N positive	Prevalence % (species with n>10 only)	Diet	Roost	Colony	Size of colony	Opportunism
**Mormoopidae**	173	19	11					
*Pteronotus rubiginosus* & *P*. sp.3*	173	19	11	ins	1	P	3	1
**Molossidae**	59	2	3.3					
*Molossus barnesi* & *M*. *molossus*	57	2	3.4	ins	4	P	2	3
*Molossus rufus*	2	0		ins	4			
**Phyllostomidae**	760	78	10.3					
*Anoura geoffroyi*	81	7	8.6	omn	1	P	3	1
*Artibeus cinereus*	1	0		fru	1	M	1	3
*Artibeus concolor*	1	1		fru	1	M	1	3
*Artibeus gnomus*	1	0		fru	1	M	1	3
*Artibeus lituratus*	3	0		fru	1	M	1	3
*Artibeus obscurus*	5	0		fru	1	M	1	3
*Artibeus planirostris*	50	6	12	fru	1	M	1	3
*Carollia perspicillata*	214	15	7.0	fru	1	P	2	3
*Chiroderma villosum*	1	0		fru	3	M	1	2
*Desmodus rotundus*	294	42	14.2	hae	2	M	2	3
*Diaemus youngi*	5	1		hae	2	P	2	3
*Glossophaga soricina*	4	1		omn	4	P	2	3
*Micronycteris hirsuta*	1	0		ins	2	M	1	2
*Phylloderma stenops*	2	1		ins	2	M	1	2
*Phyllostomus discolor*	4	0		ins	2	P	1	1
*Phyllostomus elongatus*	2	0		ins	2	P	1	1
*Phyllostomus hastatus*	1	0		ins	2	P	1	1
*Phyllostomus latifolius*	5	0	0	ins	2	P	1	1
*Platyrrhinus brachycephalus*	2	0		fru	3	M	1	2
*Sturnira lilium*	43	1	2.0	fru	4	M	1	3
*Sturnira tildae*	20	2	10.0	fru	3	M	1	2
*Tonatia saurophila*	4	0		ins	2	P	2	2
*Tracops cirrhosus*	5	0		omn	2	P	1	2
*Uroderma bilobatum*	11	1	9.1	fru	3	M	1	2
**Vespertiniolidae**	3	1						
*Eptesicus furinalis*	3	1		ins	4	M	1	3

N = number of animals

Diet: fru = frugivore; hae = haematophagous; omn = omnivorous; ins = insectivorous.

Roost: 1 = caves only; 2 = caves, old trunks; 3 = foliage; 4 = opportunist (houses, foliage, caves, trunks)

Colony: M = monospecific, P = multispecific

Size of colony: 1 = small (some animals), 2 = medium (dozens of animals), 3 = large (hundreds of animals)

Opportunism refers to the level of ecological plasticity and tolerance to perturbation: 1 (strict ecological requirements) to 3 (highly tolerant/plastic)

* the two species of mustached bats (*Pteronotus* spp.) are currently under revision and are difficult to differentiate based on external morphology only [62].

### Collection of specimens and biological material

A first set of 995 individuals (30 species) were captured on 24 sites (S1 Table) at night (from 07:00 pm to 11:00 pm) using a combination of long (12 × 2.6 m) and short (6 × 2.6 m) Japanese mist nets (Ecotone, Gdynia, Poland) on the forest floor, as implemented in the region [43]. Eight hundred and seventy-four animals were sampled for blood (between 20 and 50 μl) with a sterile needle and capillary at the brachial vein and for saliva with a swab, before release. External pressure was exerted on the vein with a sterilized absorbent hemostatic sponge to prevent bleeding and facilitate healing. Skin biopsies were also collected on the patagium using adapted small scissors for molecular identification and future genetic analyses. Blood samples were preserved at 4°C until arrival at the laboratory and centrifuged at 6500 *g* for 10 min to separate sera. Sera were preserved at −80°C and then sent for serological analyses to the NRC for Rabies, Institut Pasteur, Paris. Sixty-one vampires, collected on sites where human attacks were registered, were euthanized and organs collected (brain, heart, lung, kidney, spleen, saliva). Individuals were identified based on external morphology [44], associated with forearm measurement, and confirmed with molecular barcoding (sequencing of the cytochrome oxydase 1 and/or cytochrome b, [43]) for all species (Genbank accession numbers KU295471-KU295500).

Within the two breeding caves (Fig 1), captures were implemented every 4–5 months over a 4-year period, between Nov. 2009 and May 2013 (S2 Table). At each capture session, the population size was estimated visually by two independent observers. Captures were performed with nets, in the caves, during the day time to localize the colony at rest and target captures on vampires only, and avoid disturbing the other species. To prevent repeated disturbance of the colonies, captures were implemented by three persons only and lasted no more than 30 min. Whenever possible, females with their offspring were not captured and were directly released if they fell into the nets. All animals captured were kept in individual bags, and were identified, sampled and measured outside the caves. Seven and nine sessions of capture/recapture samplings were implemented in caves 1 and 2, respectively (Table 2), allowing marking (Passive Integer Transponder BackHome, Virbac, France, injected subcutaneously between the shoulders). Adult age was determined on the basis of completely fused phalangeal epiphyses.

**Table 2:** Individual vampire seroconversion over time. Seropositivity titers are noted for each capture. Positive seroconversions are indicated by +, negative seroconversions by—and positive seroconversions followed by seronegativations by +/-.

	Cave 1											Cave 2			
Seroconversion	+	+/-	+	+/-	+/-	+	+	+/-	-	+/-	-	-	+	+	-
Animal	Ind. 1	Ind. 2	Ind. 3	Ind. 4	Ind. 5	Ind. 7	Ind. 8	Ind. 9	Ind. 10	Ind. 11	Ind. 12	Ind. 13	Ind. 14	Ind. 15	Ind. 16
November 2009	0														
February 2010		0	0	0											
July 2010				0.6	0										
November 2010		0	0	0	1.1										
February 2011												2.5	0.9		
March 2011	0	0		0		0	0	0							
June 2011			1.5			1.2	0.9	0.8					1.3	0	1.3
November 2011	1.2	1.4			0	0.9	1.3	0	1.6	0				1.2	0
April 2012								0	1.5	1	1.1				
August 2012		0							0	0					
September 2012												0			
May 2013											0				

### Molecular screening, nucleoprotein and glycoprotein amplification, phylogenetic analysis

For molecular screening of all samples, total RNA was extracted from blood clots using the NucliSENS EasyMAG bio-robot (BioMérieux, Marcy l’Etoile, France). At the same time, for the common vampire bats only, total RNA was also extracted on swab saliva using the same protocol. cDNA was prepared with the SuperScript III Reverse Transcriptase (Invitrogen, Life Technologies, Inc.) and random hexamers (Roche, Mannheim, Germany). Presence of lyssavirus was investigated using a hemi-nested RT-PCR (hnRT-PCR) that has been described to amplify a wide range of lyssaviruses [45]. This hnRT-PCR amplifies a fragment of 249 nucleotides of the viral polymerase (L) gene. Amplification products at the expected size were confirmed by sequencing (Beckman Coulter Genomics, Takeley, UK).

The nucleoprotein (N) and glycoprotein (G) sequences obtained from the RABV-positive sample originating from a common vampire bat were obtained using a nested RT-PCR approach, after total RNA extraction from heart and cDNA synthesis, as described above. For the N gene, the first round of PCR was performed using primers 21G and 304S, as described in [35]. The nested PCR was performed using the forward primer RABV 719F (5′-ATTGAACATTTGTATTCAGC-3′) and the reverse primer RABV 827R (5′-CAGTGAGATTTATTTGCTTT-3′) designed in this study. To generate the complete sequence of the G gene, two sets of primers (umf2/994b and 760f/308b) amplifying two overlapping fragments were used in the first-round PCR as previously described [46]. Then, the initial umf2/994b PCR product was used as the template in a nested PCR using the forward primer RABV 3734F (5′- AAATCCTTATCCTGATTACCAC -3′) and the reverse primer RABV 3876R (5′-CCAAACATTTCCCACCAGGGAACACC-3′), while the initial PCR product760f/308b was used as template in a nested PCR using the forward primer RABV 4717F (5′-GAAGTCCACCTCCCAGATGTTC-3′) and the reverse primer RABV 5044R (5′-GCAGTCTTCAGATCGCAGGATA-3′). Amplification products of the expected size were then sent for sequencing to Beckman Coulter Genomics (Takeley, UK). In addition to the samples analysed in this study, data obtained independently by the NRC for Rabies in its diagnostic and surveillance activities on moribund mammals were included in the analyses. Nine domestic animals (two dogs and seven cattle species) and one bat (*Artibeus planirostris*), collected in French Guiana between 1989 and 2009 were added. The N gene was amplified and sequenced for the ten animals and the G gene was only generated for the bat sample as previously described [47–49].

The two genomic regions (N and G complete gene sequences) were analysed separately. Raw sequences were analysed and edited in MEGA 5.05 [50]. Sequences were translated into amino acid and both nucleotide and amino acid sequences were checked for irregularities. Multiple sequence alignments were performed using MEGA 5.05 with other previously published rabies virus sequences, and alignments were checked manually. Phylogenetic trees were inferred from the aligned nucleotide sequences. The MrModeltest2.3 program [51] was used to determine the optimal model of nucleotide evolution for the two genes. The GTR model, with a gamma distribution shape parameter (G) and invariable sites (I), was identified and used for the Bayesian approach, which was performed with Mr. Bayes 3.2.2 to infer phylogenetic relationships [52]. Markov Chain Monte Carlo (MCMC) simulations were run for 10,000,000 generations, with four simultaneous chains, using a sample frequency of 100 and a burn-in of 25,000. Majority rule consensus trees were obtained from the output. Validation of the inference was assessed based on the standard deviation of split frequencies, which was less than the expected threshold value of 0.01 (calculated value of 0.0018 and 0.0009 for the N and G genes, respectively).

### Serologic methods

The technique used to detect RABV neutralizing antibodies is the Rapid Fluorescent Focus Inhibition Test (RFFIT) [53]. A constant dose of a previously titrated (calibrated to give 80% fluorescent focus infected cells) cell culture-adapted RABV challenge virus (CVS) was incubated with threefold dilutions of the sera. After incubation of the serum-virus mixtures, a suspension of a clone of BHK-21 (BSR) cells was added. After 24 h of incubation, the cell monolayer was acetone-fixed and labelled with a fluoresceinated anti-nucleocapsid antibody (Bio-Rad, Marnes-la-Coquette, France). The optimal challenge dose (the dilution giving 80% infected cells for each virus production) was calculated. Titers of sera were calculated by comparison with a reference serum calibrated to the WHO reference serum. A minimal threshold of 0.5 IU/ml was considered for positivity. This titer is close to the positivity thresholds used in studies conducted on bats [11, 54–56] or other non-flying wild mammals [57–59] and is conservative and therefore further increases the specificity of the test.

### Statistical analysis

Ecology and biology extensively vary among species (Table 1). To control the bias of unrelated sample size for each species at each capture site rather than specific prevalence, we used individual serological response. A multivariate logistic regression analysis was used with R [60] to infer the role of bioecological factors including diet, type of roots, type of colonies (monospecific *vs*. multispecific), size of colonies, and ecological plasticity [44,61], as well as the characteristics of capture sites, including vegetation type [41], landscape units [42] and disturbance index [40]. For vampire bats that were recaptured, only the serological result obtained at their first capture was considered. To limit the uncovered bias of likely temporal and geographic variations of serology (*e*.*g*., [22], this study), we considered the year of sampling and the capture sites as random effects. A two-step procedure was used in the logistic regression model to determine the most parsimonious multivariate model. First, the association of all factors and the positivity were assessed one by one in bivariate analyses with the year as random effect; a *p*-value < 0.1 allowed selecting candidate variables for the second step. In this second step, the same logistic regression model including all factors identified in the first step was applied, with a generalized linear mixed model fit by maximum likelihood. The best multivariate model was selected with AIC and BIC criteria.

## Results

### Molecular detection of RABV and phylogenetic relationships

The molecular survey realized on 995 healthy bats captured between 2005 and 2013, identified one positive common vampire bat *Desmodus rotundus* for RABV (Table 1). This animal was a female adult, not pregnant, that was captured in September 2010, on the Approuague River, North-East of French Guiana. RABV RNA was detected in blood, saliva and heart tissue (the brain was not tested). This animal was also positive by serology, with a low titer (0.6 IU/ml). Sequences of this RABV-positive sample (AT6) were obtained for the G and N genes and deposited in GenBank (accession numbers KT023100 and KT023101, respectively). Furthermore, the only other bat (*Artibeus planirostris*, specimen 09035FRA) detected as positive by the NRC for Rabies was found in a sublethal condition in Rémire-Montjoly, close to Cayenne in 2009. All the sequences obtained by the NRC were deposited in GenBank (accession numbers KT276358 and KT276360 to KT2763688 for domestic species; KT276359 for the *Artibeus*).

**Fig 2:**
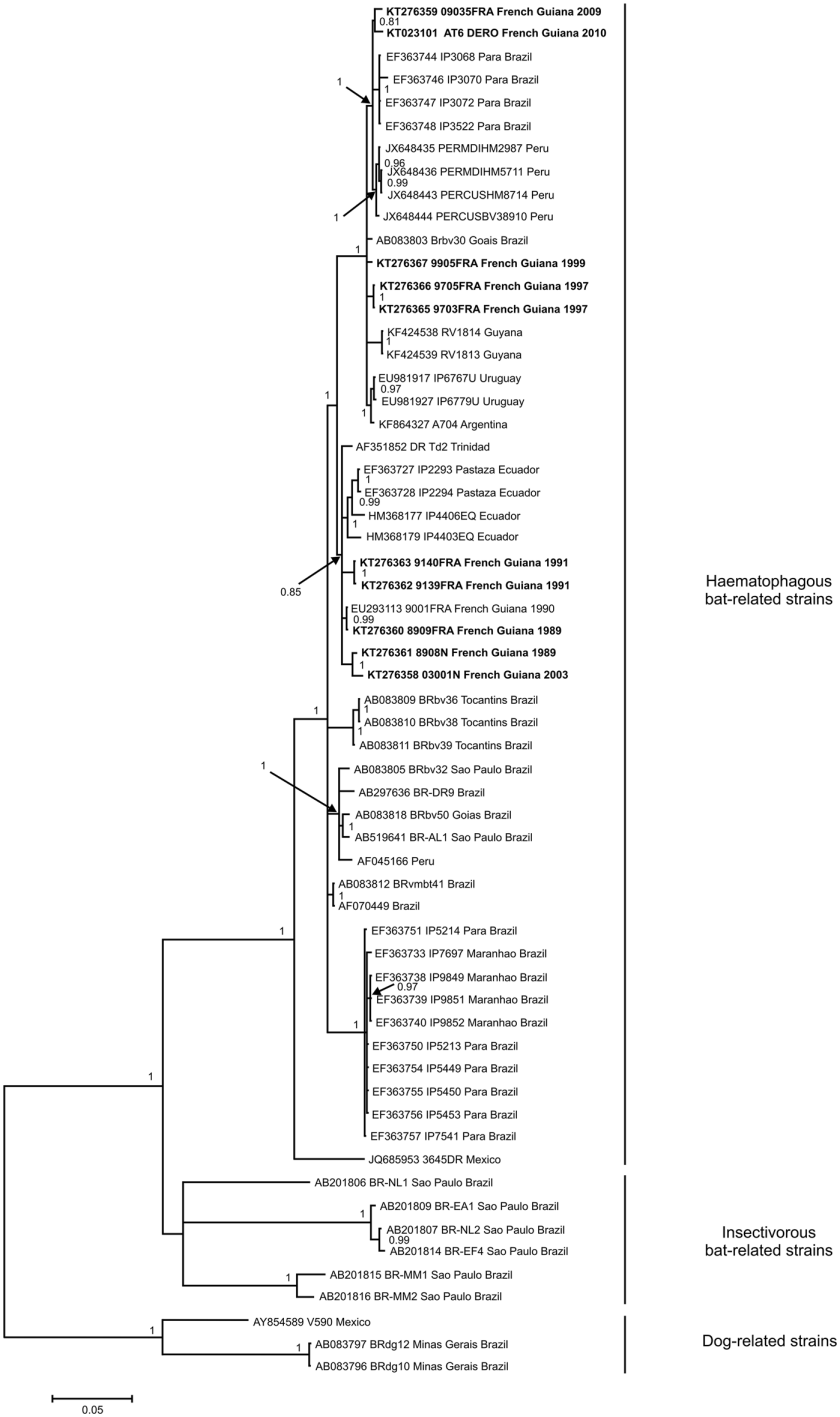
Phylogenetic tree constructed using Bayesian methods based on 1314 nucleotides of the nucleoprotein gene. Virus names are associated with their accession numbers. Novel sequences generated from the two rabid bats in this study are shown in bold. Support for nodes is provided by the posterior probabilities of the corresponding clades. All resolved nodes have a posterior probability greater than 0.8. Scale bar indicates nucleotide sequence divergence among sequences.

**Fig 3:**
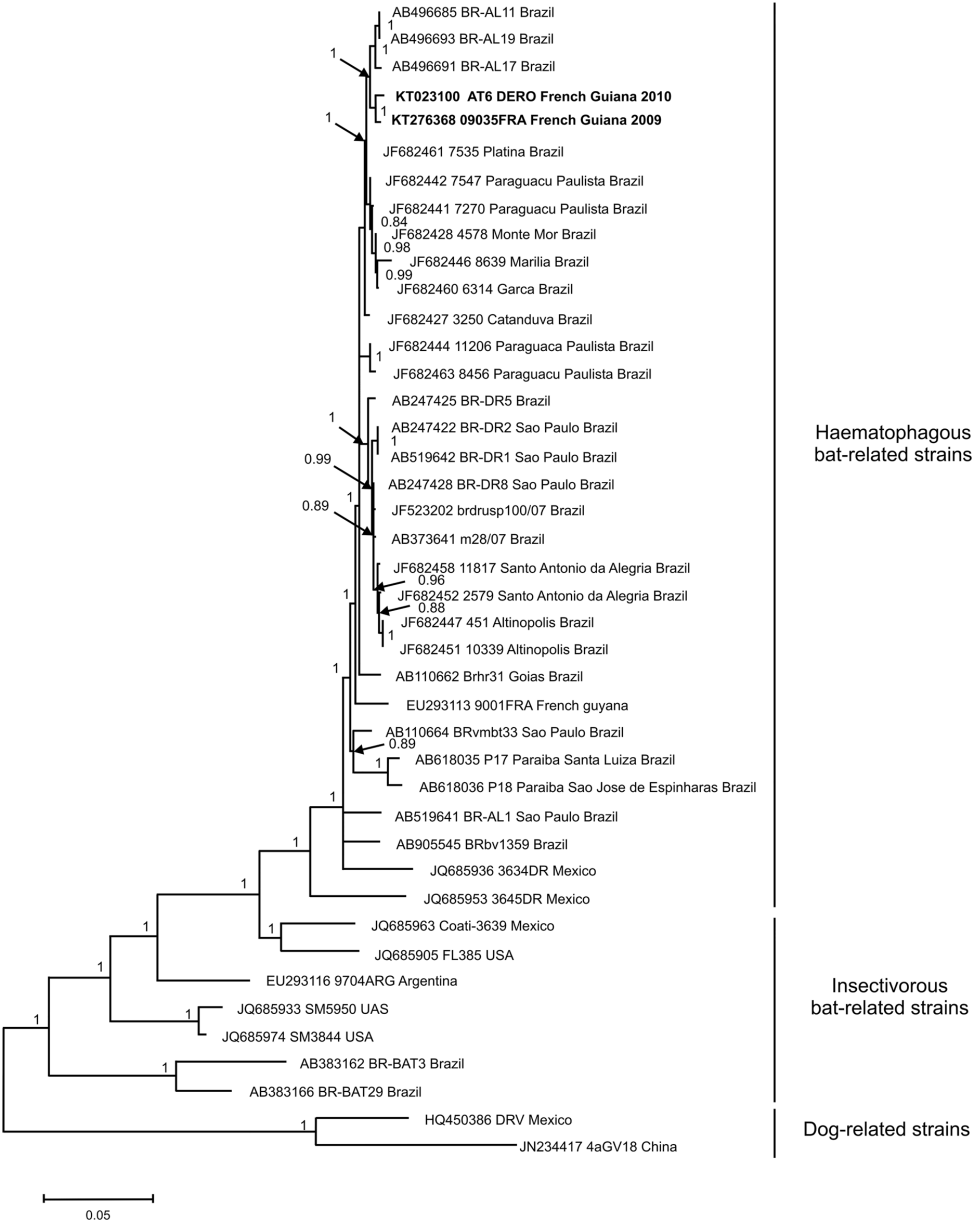
Phylogenetic tree constructed using Bayesian methods based on 1572 nucleotides of the complete glycoprotein gene. Virus names are associated with their accession numbers. Novel sequences generated from the two rabid bats in this study are shown in bold. Support for nodes is provided by the posterior probabilities of the corresponding clades. All resolved nodes have a posterior probability greater than 0.8. Scale bar indicates nucleotide sequence divergence among sequences.

For the N gene, all sequences isolated from bats, dogs and cattle in French Guiana belong to the haematophagous bat-related strains. They cluster in three different groups according to their isolation date. The first group, composed of strains isolated between 1989 and 2003, shows a high percentage of sequence identity within the group (between 98.5% and 100%). They are related to strains identified in Trinidad and Ecuador between 1995 and 2007. The second group of sequences is made up of strains isolated between 1997 and 1999. They show between 99.5 and 100% identity within the group. These strains have a basal position within the clade to which the two sequences identified from bats (AT6 and 09035FRA) belong. Sequences of AT6 and 09035FRA show a high percentage of identity (99.3% in nucleotide) within the group and with a group of haematophagous bat-related viruses identified in Brazil (Pará state) and Peru (99.8% and 99.3%, respectively). Phylogenetically, the most closely related sequences to the two strains identified in French Guiana in 2009 and 2010 are from Peru and the Pará state in Brazil and represent a clade circulating in the North of the Amazonian region (Fig 2). For the G gene, sequences obtained from *Desmodus rotundus* AT6 and from *Artibeus planirostris* 09035FRA show between 98.9 and 99.2% identity with Brazilian strains. Furthermore, they show 97.4 and 97.6% identity, respectively, with strain RABV 9001FRA isolated in French Guiana in 1990 [63]. These sequences are phylogenetically related to G sequences from strains identified in Brazil and isolated from frugivorous bats (*Artibeus lituratus*) (Fig 3). They all belong to the haematophagous bat-related clade.

### Serological evidence of RABV circulation, biological and ecological factors influencing seroprevalence

Nine hundred and ninety-five bats (including those captured during vampire monitoring) belonging to 30 species were sampled and analysed to investigate the environmental and bioecological factors that could be related to variation in seroprevalence (Table 1).

Positive animals were recorded in 14 out of the 30 species analysed, with specific seroprevalence ranging up to 14% for the vampire species (seroprevalence rates were only calculated for species with n>10 animals) (Table 1). At the family level, Phyllostomidae had a seroprevalence rate of 11.3%, Mormoopidae 11%, and Molossidae 3.3%. Nevertheless, sample sizes showed significant differences, both in terms of the number of samples collected and species richness within these families, thus precluding any relevant conclusions.

Overall, no difference was recorded between genders (10.5% in females *vs*. 9.9% in males, chi^2^ = 0.08, df = 1, *p* = 0.78) and both genders also exhibited the similar mean titer (1.0 ± 0.1). No difference was recorded between seasons: 11.9% and 11.7% during the dry and wet season, respectively (chi^2^ = 0.01, df = 1, *p* = 0.92). The univariate analysis identified diet (haematophagous *vs*. other types of diet, *e*.*g*., omnivorous, frugivorous, insectivorous), type of roosts (generalists species *vs*. those using specific roosts only such as caves, leaves, trunks), monospecific colonies and forest vegetation as factors favouring positivity. Selecting these factors only for the multivariate logistic regression analysis, two models of comparable relevance were identified. The first model (AIC = 595, BIC = 624) considered diet (haematophagous *vs*. other diets, *p* = 0.003, OR = 2.15) and vegetation classes (forest habitats *vs*. disturbed and open areas, *p* = 0.0084, OR = 2.0). The second model (AIC = 598.49, BIC = 626.00) considered the structure of the colonies (monospecific *vs*. multispecific, *p* = 0.007, OR = 2.1) and the vegetation classes (forest habitats *vs*. disturbed and open areas, *p* = 0.003, OR = 2.3). Considering these two models, haematophagous diet, monospecific colonies and dense forest habitats favour rabies virus seropositivity.

### Temporal variations of serology in the *Desmodus rotundus* vampires

In both caves, repeated visual estimations by two independent observers suggest that the size of the population ranges from 60 to 100 individuals in cave 1 and from 120 to 150 individuals in cave 2. Three hundred and one unique animals were captured over a 4-year period (144 animals in cave 1 and 157 in cave 2), including 279 adults and 22 juveniles (forearm < 55 mm). Among those animals, 33 were recaptured at least once (22 animals in cave 1 and 11 in cave 2) and up to five recaptures were noted for one animal (Table 2). No recapture was observed between the two caves located 25 km apart.

**Fig 4:**
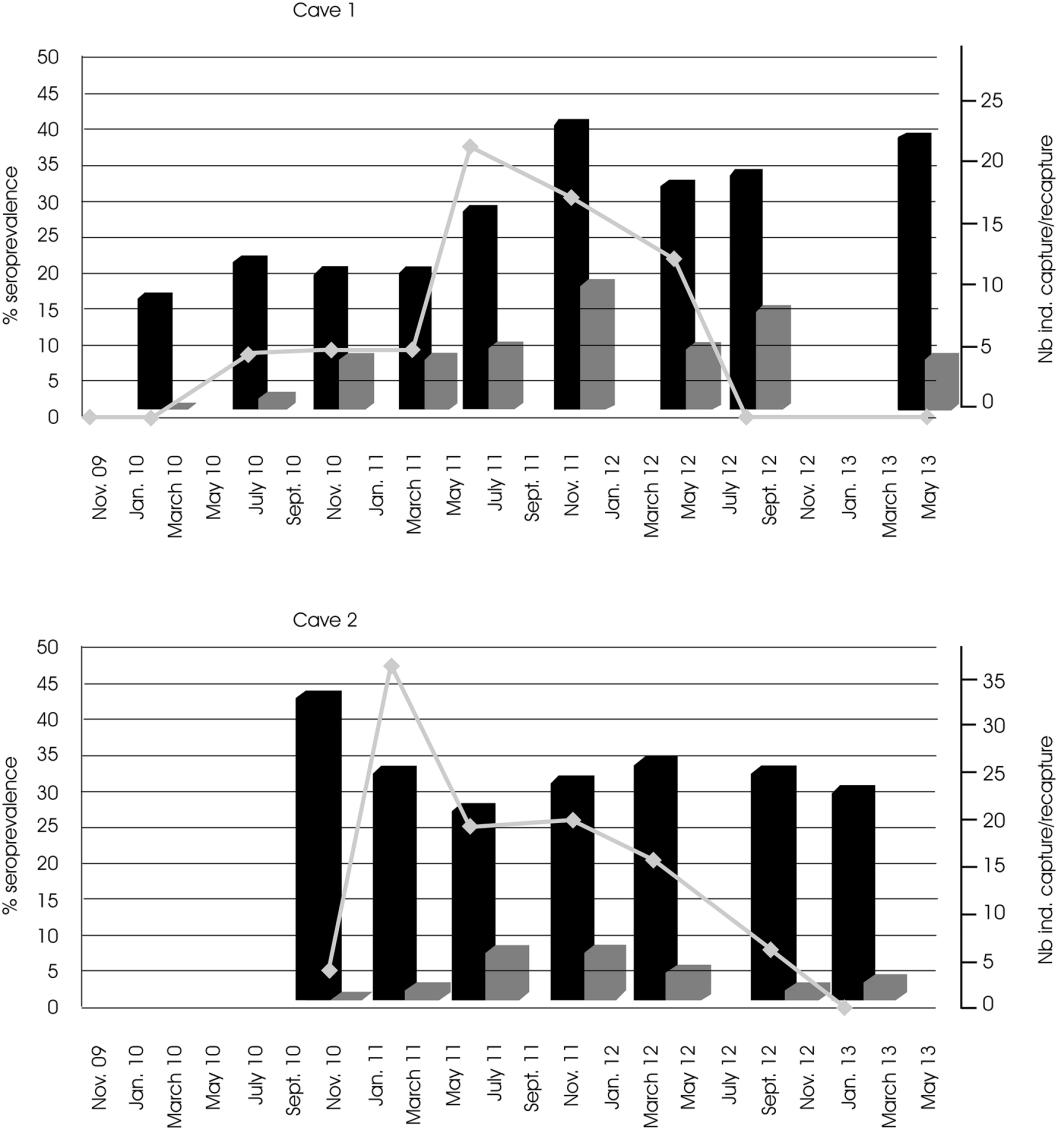
Changes in the percentage of RABV seropositive vampire bats from November 2009 to May 2013 in the two caves and the number of animals captured and recaptured during the same period. Grey line: % of seropositive animals. Black bar: total number of animals captured. Grey bar: number of recaptured animals.

Pooling all juveniles showed an overall prevalence of 4.1% *vs*. 15% in adults but the low sample size of juveniles precludes detailed investigation and comparison related to age. During this longitudinal study, the first 2-year period showed an increase in the number of recaptured animals, as expected in a closed population, and then fewer captures of previously identified vampires, followed by a new increase. This is indicative of a partial departure of the population present during the first two years and the arrival of a second pool of individuals (Fig 4). Seroprevalence rates varied over time, from 0% to 38% to 0% (cave 1) and from 0% to 47% to 0% (cave 2), and almost concomitantly in the two caves (Fig 4). The increase and decrease in seroprevalence rates were concomitant with the increase and decrease of the recaptured/captured (*i*.*e*. new animals) ratio, but no mortality was observed.

At the individual level, on the basis of the 33 different animals recaptured, 18 animals were seronegative each time. We observed a positive seroconversion (seronegative to seropositive antirabies status) in six animals, a negative seroconversion in four, while five animals had a positive seroconversion followed by a negative one 6–12 months later (Table 2).

## Discussion

In South America, numerous studies have investigated the circulation of RABV through molecular and serological surveys but only a few have focused on wild bat populations in the Amazonian forest region. Combining these two approaches, we conducted (i) a large survey on a wide diversity of habitats, characterized by different levels of specific richness in bat communities [7,64] and (ii) temporal monitoring of vampire colonies. This survey provides new insights into the distribution, circulation and dynamics of the RABV in bats communities from the Amazonian forest region.

### High diversity of hosts

The survey reported herein increases the diversity of bat species explored for RABV infection in Amazonia and it demonstrates that a large set of species exhibit antibodies, some of which have not been evidenced before [2,29] such as, *Phylloderma stenops*, *Pteronotus rubiginosus* and *Sturnira tildae*. As can be expected [5], the common vampire *Desmodus rotundus* is the species with higher seroprevalence rates (19.3% in our study), but important differences were noted among sites and time periods. These results are in agreement with those previously reported in several countries such as Trinidad, Grenada and Peru [9,65]. Nevertheless, these rates are far lower than those observed in the Eastern Brazilian Amazon area with values ranging from 38 to 59% depending on the capture sites, which were located near cattle ranching [12]. The particular location of these sites could explain the high seroprevalence observed in these areas where transmission of rabies can be favoured by high vampire bat densities and their concentration around cattle, which may favour increased interactions between animals.

From a phylogenetic viewpoint, all RABV sequences of the N gene detected in French Guiana belong to the clade of haematophagous bat-related strains. Within this clade, two rabies lineages are circulating in French Guiana: (i) one with strains associated with Ecuadorian strains and (ii) the other one with strains from Brazil and Peru. In the latter clade, the N sequences obtained from the two positive bats (*Artibeus planirostris* and *Desmodus rotundus*) fall within "lineage II" of sylvatic rabies strains that circulate within a large geographic range in South America [13]. Indeed, this lineage includes isolates originating from Peru (Cusco department in 2003, Madre de Dios and Puno departments during a human rabies outbreak in 2007), Brazil and Uruguay identified between 2004 and 2008, and now from Northern Amazonia. Furthermore, strains of this lineage could be dispersed *via* several species, such as *Artibeus* and *Desmodus* [35, 66]. Analysis of the G gene also shows that our RABV sequences are phylogenetically closely related to Brazilian strains identified in *Artibeus lituratus*.

Cross-species transmissions have been described to occur in phylogenetically closely related species and, to a lesser extent, have been related to ecological closeness [8]. In French Guiana, only the haematophageaous clade has been evidenced, and no strain related to the insectivorous lineages (Fig 3) has been identified, although insectivorous species have been detected as positive in serology.

### Factors influencing infection: Importance of habitat and community richness

We observed considerable variations of the seroprevalence rates according to species. The multivariate approach identifies three risk factors for bat infection. The first one is related to habitat, while the two others are related to the bioecology of the species. Forest habitats are associated with a significantly higher risk of positive serology; a similar result was observed in Mexico and attributed to more frequent interspecies interactions in areas of higher species richness [67]. In another Amazonian region, although regional differences have been recorded, no habitat pattern was detected [12]. With the current knowledge of RABV ecology, it is difficult to propose a hypothesis explaining the contribution of the vegetation type in the circulation of the virus. Nevertheless, given the structure of bat communities, with their diversity and richness influenced by forest types and level of habitat disturbance [68], one can envision that the spread of the virus lineages is related to the diversity and dynamics of bat populations and possible spill-overs among species. In agreement with this hypothesis, bats with a more fragmented distribution in South-East Asia were shown to have a lower viral richness [69]. In addition, considering the lower richness of bat communities in disturbed areas, one can observe a lower viral circulation, possibly related to geographic characteristics, which can affect bat dispersal, and transmission of the virus between bat species and colonies [70]. Also, edge effects tend to reduce the diversity of habitats, implying a reduction in the diversity of ecological niches, both for hosts and microorganisms.

The diversity of hosts raises the question of transmission of the virus between individuals from the same species and between species. In the case of multispecific roots, such as the vampire and the mustached bats (*Pteronotus rubiginosus* and *P*. sp. 3), syntopy in caves could explain the high seroprevalence rates observed. Nevertheless, other species, such as the frugivorous *Artibeus*, with completely distinct ecological requirements, are also infected.

As previously mentioned, cross-species transmission between different bat species in the Americas seems to be correlated with phylogenetic distance between species and to a lesser extent to ecological factors [8]. However, contacts with heterologous rabies variants among bats could induce seroconversion even in the absence of infection, thus impacting the seroprevalence observed. In French Guiana, the bioecological diversity of species exhibiting antibodies suggests that contacts with heterologous rabies variants occur in all forest strata, in most habitats and between species that do not share the same microhabitat and are not in syntopy. The routes and mode of rabies virus exposure between bat species require further investigation, even between phylogenetically closely related species that may use different roosts and/or habitat (canopy, undergrowth, etc.). The possible contacts with rabies virus disseminated in aerosols (presence of rabies in lungs) and/or by other routes of excretion in urine and feces (virus identified in kidneys and stomach) could be suggested [71,72]. Nevertheless, seropositivity can also result from sporadic spillover among different species. Thus, the presence of RABV antibodies in different bat species sharing the same space should not be interpreted as circulation of the virus among these species and does not confirm a reservoir role for them *per se*.

The high prevalence rates observed in forest communities associated with a rather short period of detectable antibodies (see below) suggest frequent infections and re-infections. The analysis of ecological factors that might affect the infection dynamics observed in European insectivorous bat colonies infected by another lyssavirus, the European bat lyssavirus type 1 (EBLV-1), demonstrated that the seroprevalence was significantly associated with colony size (similar to our study) and species richness (unlike our study). Indeed, the higher seroprevalence percentages were found in large multispecific colonies, suggesting that intra- and interspecific contacts are major risk factors for EBLV-1 transmission in bat colonies [73].

### Temporal variation of seroprevalence in vampires: Enzootic *vs*. epizootic cycles?

The temporal monitoring of vampire communities contributes new information since the pioneering work [22]. As shown in Peru, we observed considerable temporal variation of seroprevalence, but we did not link this variation to any seasonal or climatic factor. Also, unlike this first study in Peru, we observed that colonies are not stable over time. We cannot exclude a temporary abandonment of the sampled caves because of the disturbances caused by our study, even though we tried to limit the time spent in the caves (the youths were released immediately, we limited light and number of persons for captures, and all samples were collected outside the caves). More likely, however, increases and decreases of recaptures and seroprevalence rates in the two caves argue in favour of both dispersal and movement of individuals between vampire bat colonies and periodic reintroduction of RABV among colonies [73] (Fig 4). In contrast, the high number of apparently healthy and seropositive bats indicates that, at least in a large number of cases, the clinical expression of the virus, if any, is undetectable. Furthermore, some vampires proved to seroconvert during the monitoring period in both ways (Table 2). The question of virus control by the host and/or the ability of the virus to persist in the host need to be further investigated. Finally, high and fluctuating seroprevalence rates and high level of circulation of the virus among individuals, populations and species support an enzootic hypothesis once introduced in a new area [22]. Another hypothesis that seems, until now, less likely is that the virus persists in the host, escaping the immune response, thanks to reactivation phases induced by physiological, biological and ecological constraints. Few other studies have addressed the inter-annual dynamics of lyssaviruses among bat multispecies that are roosting in the same refuge and relate similar fluctuations in the percentage of seropositive bats observed and survival of the bats during the time of the study [56,74,75]. These longitudinal and temporal surveys demonstrate that cyclic lyssavirus infections occur with periodic oscillations, likely controlled by the number of immune and infected bats, and the variable persistence of immunity depending on the host species. These questions on virus control and persistence warrant further investigation.

### The emergence risk: Importance of the conservation status of habitats and bat communities

Emergence of viral diseases, including rabies, is classically linked to pressures and threats on natural habitats. Higher seroprevalence rates in areas with cattle ranching attributed to disturbed or deforested areas were evidenced in the Amazonian region [12]. The highest seroprevalence rates we observed in bats living in forest habitats, similar to observations in Mexico [67] corresponding to more preserved areas, contradicted the earlier results reported in Amazonia [12]. In French Guiana, ranching activities are very scarce, with limited deforestation and few edge effects. We therefore hypothesize that, in areas with well-preserved habitats, RABV circulation is mainly maintained in forest areas where food is available for the main prevalent reservoir species (*Desmodus*). This particular context in French Guiana, with no or few human impacts except on littoral areas, could be considered as a “basal” situation of rabies virus circulation in vampire and other sylvatic bat species. This situation supports an "amplification effect" hypothesis (as described by [76]), which predicts that diversity will increase the circulation of the virus, and underlines the relevance of different ecological factors in determining infection risk in bat populations under different levels of anthropic influence.

Contrasting with other Amazonian and neotropical regions where rabies emergence phenomena are reported (Peru, Ecuador, Mexico) and despite a growing human population, the Northern Amazonian region still faces low deforestation rates. Over the last decade (2000–2012), forest loss was less than 0.2% in French Guiana *vs*. 1.8% for the Peruvian Amazonia region, 1.4% for Ecuadorian Amazonia and 1.2% for Mexico (data derived from [31]). The good conservation status of forest habitats in French Guiana, and more widely in the region (Suriname, French Guiana, the Brazilian state of Amapa), low human density, rather favourable economic conditions limiting highly unhealthy living conditions, and efforts to provide post-exposure treatment for most of the population [37] likely explain the low to very low occurrence of human rabies cases. Long before spectacular deforestation, human activities may induce the more cryptic and insidious defaunation effect, with a cascade of ecological consequences [77]. One of these is the disappearance of resources for tertiary or secondary consumers, levels in the trophic chain that can include vampires. As predators, populations of vampires that would face quick disappearance of primary resources would have to shift to alternative resources, which in edge habitats could be cattle, domestic animals and humans, with associated crossing of the species barrier by the virus. Comparative use of stable isotopes or blood-meal PCR analyses in both forest and edge populations could help to support this hypothesis. A good forest status, allowing a dilution effect (*sensu* [78]) in highly rich bat communities and the maintenance of large populations of medium-sized and large mammals used as preys by haematophagous bats, should prevent their migration to anthropized areas. However, at a very local scale, even in a well-preserved country, vampire attacks are sometimes recorded in forest settlements such as tourist camps and small Amerindian villages ([37]; A. Lavergne & B. de Thoisy, pers. obs.). These attacks are likely the response to local defaunation processes such as overhunting [79], destruction of a colony’s habitat or ecological opportunities such as a human settlement with its associated biomass of mammals. The risk of rabies virus transmission must nevertheless be adequately evaluated. Indeed, two bats of the approximately 1,000 tested (for vampires only, one positive animal out of 261) were positive for RNA and consequently had the potential for spreading and transmitting RABV.

With the identification of an increasing number of bat species potentially implicated in rabies epidemiology, the risk of interaction between bats, livestock, humans and domestic animals must more than ever be considered and further studies are necessary to clarify the role of not only haematophagous bats, but also sympatric and syntopic species. In addition, the role of environmental variables in virus circulation and persistence in hosts and colonies requires further attention. Without a full understanding of the biology of rabies virus in its reservoir, only transversal approaches on ecological, evolutionary, immunological and virological aspects will shed light on infection outcome and disease tolerance in bats and on the risk of transmission to other host species and humans.

## Supporting Information

S1 Table Study sites for bat sampling, French Guiana. (DOCX)

S2 Table Sampling dates of common vampire bats for capture / recapture monitoring. (DOCX)
