# Supplementary material for: Bioecological Drivers of Rabies Virus Circulation in a Neotropical Bat Community
Source: PLoS Negl Trop Dis. 2016 Jan 25;10(1):e0004378. doi: 10.1371/journal.pntd.0004378 (PMC4726525; doi:10.1371/journal.pntd.0004378)
Supplement: S1 Table — (DOCX) [file pntd.0004378.s001.docx]

Supplemental Table S1. Study sites for bat sampling, French Guiana

| **Sampling sites** | **location (lat/long, d,dd)** | **vegetation 1** | **biogeography** | **disturbance** |
| --- | --- | --- | --- | --- |
| 1. Grotte Parfum | 4.06 / -52.64 | 19 | 15 | 0 |
| 2. Grottte Caiman | 4.56 / -52.18 | 19 | 10 | 4 |
| 3. Grotte Mathilde | 4.54 / -52.17 | 19 | 10 | 4 |
| 4. Grotte fourgassier | 4.61 / -52.17 | 19 | 10 | 4 |
| 5. Cacao | 4.57 / -52.46 | 23 | 9 | 20 |
| 6. Roura | 4.73 / -52.32 | 21 | 3 | 25 |
| 7. Athanase | 4.23 / -52.24 | 18 | 16 | 8 |
| 8. Trésor | 4.56 / -52.27 | 19 | 10 | 4 |
| 9. Paracou | 5.23 / -52.94 | 19 | 7 | 20 |
| 10. Rémire Montjoly | 4.89 / -52.29 | 22 | 3 | 32 |
| 11. Lac américain | 4.84 / -52.34 | 21 | 3 | 25 |
| 12. Paramana | 4.81 / -52.33 | 22 | 3 | 30 |
| 13. Camp Tigre | 4.91 / -52.29 | 22 | 3 | 32 |
| 14. Bourda | 4.94 / - 52.27 | 22 | 3 | 32 |
| 15. Pasteur | 4.93 / -52.33 | 22 | 3 | 40 |
| 16. Saint Georges | 3.89 / -51.81 | 20 | 16 | 30 |
| 17. Apatou | 5.16 / -54.32 | 20 | 5 | 15 |
| 18. Molokoi | 4.55 / -52.42 | 19 | 10 | 20 |
| 19. Montsinery | 4.89 / -52.51 | 23 | 3 | 25 |
| 20. Roura | 4.72 / -52.32 | 21 | 3 | 25 |
| 21. Stoupan | 4.77 / -52.34 | 21 | 3 | 25 |
| 22. Ineri | 4.32 / -52.15 | 21 | 4 | 30 |
| 23. Chaumière | 4.88 / -52.36 | 22 | 3 | 32 |
| 24. Macouria | 4.97 / -52.49 | 23 | 3 | 25 |

Vegetation : categories are based on canopy reflectance [41].

Biogeography : categories are based on landscapes and geomorphology units [42].

Disturbance index: the continuous variable is based on index footprint method [40].
