# Supplementary material for: Bioecological Drivers of Rabies Virus Circulation in a Neotropical Bat Community
Source: PLoS Negl Trop Dis. 2016 Jan 25;10(1):e0004378. doi: 10.1371/journal.pntd.0004378 (PMC4726525; doi:10.1371/journal.pntd.0004378)
Supplement: S2 Table — (DOCX) [file pntd.0004378.s002.docx]

Supplemental Table S2. Sampling dates of common vampire bats for capture / recapture monitoring.

|  | Cave 1 | Cave 2 |
| --- | --- | --- |
| Collection dates |  | - |
|  | 06/02/2010 | - |
|  | 24/07/2010 | - |
|  | 27/11/2010 | 06/11/2010 |
|  | 23/03/2011 | 20/02/2011 |
|  | 10/06/2011 | 18/06/2011 |
|  | 20/11/2011 | 26/11/2011 |
|  | 29/04/2012 | 18/03/2012 |
|  | 10/08/2012 | 08/09/2012 |
|  | 18/05/2013 | 27/01/2013 |
|  | - | 03/09/2013 |
